# Content Validation of an Instrument for the Assessment of School Teachers’ Levels of Knowledge of Diabetes through Expert Judgment

**DOI:** 10.3390/ijerph17228605

**Published:** 2020-11-19

**Authors:** Trinidad Luque-Vara, Marta Linares-Manrique, Elisabet Fernández-Gómez, Adelina Martín-Salvador, María Angustias Sánchez-Ojeda, Carmen Enrique-Mirón

**Affiliations:** 1 Department of Nursing, Faculty of Health Sciences, Melilla Campus, University of Granada, Calle Santander s/n, 52001 Melilla, Spain; triluva@ugr.es (T.L.-V.); mlinar@ugr.es (M.L.-M.); ademartin@ugr.es (A.M.-S.); maso@ugr.es (M.A.S.-O.); 2 HUM-613 Research Group, Department of Inorganic Chemistry, Faculty of Health Sciences, Melilla Campus, University of Granada, Calle Santander s/n, 52001 Melilla, Spain; cenrique@ugr.es

**Keywords:** content validity, expert judgment, Fleiss’ *κ*, type 1 diabetes, schools, questionnaire

## Abstract

The objective of this study was to describe the content validation, through expert judgment, of a questionnaire for determining the level of knowledge that school teachers have of diabetes in order to design relevant educational interventions to improve the health of school-aged children. This psychometric study involved 15 experts who assessed each of the items in the instrument. The results revealed that the strength of agreement shown by the questionnaire ranged from substantial to almost perfect in its four dimensions, with the characteristics of “sufficiency” and “relevance” having the highest scores (0.982 and 0.903, respectively) based on the judgments made by the participating experts. Regarding statistical significance, the characteristics “sufficiency”, with *p* < 0.001, and “relevance”, with *p* = 0.001, were particularly relevant. The overall degree of understandability for the new version of the instrument was high (91.54%). The psychometric results obtained from validation of the “grado de conocimientos sobre diabetes en docentes del ámbito escolar” (Spanish for “level of knowledge of diabetes in school teachers”)—GCDDaE questionnaire through expert judgment and the pre-test indicate that it is recommended for use as it is both relevant and quick and easy to administer.

## 1. Introduction

Diabetes is one of the most common metabolic disorders in the world. In 2019, there were 578 million people with diabetes globally, which is expected to grow to 700 million by 2045 [1]. Diabetes is associated with levels of morbidity and mortality that result in a major public health problem and significant economic expenditure for both families and national economies [2]. According to the International Diabetes Federation, approximately 1,110,100 children and adolescents have type 1 diabetes mellitus (DM1), with 98,200 newly diagnosed cases each year. This figure rises to 128,900 when the age range is under 20 years, with significant regional and national differences in the number of children and adolescents (aged 0–14) with prevalent (existing) and incident (newly diagnosed) DM1 [3]. In Spain, epidemiological studies on DM1 in children under 15 years of age have revealed a mean incidence of 17.69 cases per 100,000 inhabitants per year [4].

The criteria established in the “Standards of Medical Care in Diabetes” by the American Diabetes Association [5] mention that schools must ensure that children with DM1 have a safe environment that allows for proper control of the disease and thus facilitate their full participation in school and extracurricular activities. In this regard, it is essential that teachers have a minimum knowledge of the management of the disease, particularly in the event of an emergency, in order to improve the quality of life of children with diabetes and avoid long-term complications [6].

There is also a need for health measuring instruments that may be used in clinical and research practice. These instruments must undergo a validation process, which is a prerequisite to being able to ensure their quality [7,8].

Research on the validation process as well as on the construction and adaptation of measurement instruments is insufficient in the field of health, and information is often fragmented [9].

According to Galicia et al. [10], there are different types of validation methods, including construct validity, criterion validity, and content validity, with construct validity being the most widely used. In this study, content validity was the validation criterion used.

Content validity may be defined as “the degree to which elements of the measurement instrument are comprehensive, relevant, and representative of the construct for a particular assessment purpose” [11] (p.238). As a result, higher levels of content validity in an instrument are indicative of higher levels of precision in the measurement of the target construct [12]. For a measurement instrument to be considered to be valid, it must be simple and affordable, free of bias, appropriate for the problem under study, have clearly defined dimensions, and adequately reflect the concept that it is intended to measure [13].

In addition, instruments must have two essential characteristics of good quality, i.e., validity and reliability, so that they can be used by researchers in their studies [14,15] while ensuring the integrity of their results [12].

The usual procedure for assessing the content validity of the instrument is to consult experts [16]. According to Escobar-Pérez and Cuervo-Martínez [17], expert judgment may be defined as an informed opinion from individuals with a track record in the field, who are regarded by others as qualified experts in the field, and who can provide information, evidence, judgments, and assessments (p. 29). The way these judges are chosen is thus fundamental, since they must be experts in the field, either because of their academic training or because of their work experience. Once selected, the experts make an assessment of the different dimensions of the questionnaire using a numerical scale as part of the procedure [10]. According to Fernández-Gómez et al. [18], the said assessment should incorporate both qualitative and quantitative data. The process that judges undertake becomes a fundamental effort to eliminate unimportant aspects, modify aspects that need to be modified, and incorporate relevant aspects [15]. The researcher will then analyze this process thoroughly, and will determine what to modify, improve, or remove from the instrument to be validated [10].

With this technique, it is possible to calculate its content validity index, with the adoption of rigorous statistics and an appropriate methodology, so that the instrument assessed may be used for the purpose for which it was designed [19].

Today, an increasing number of school children have chronic health problems, diabetes is one of the most prevalent conditions affecting this group [20]. Given that diabetes at a young age requires constant care in both family and school settings [21] and that, according to a number of authors [22,23,24,25,26,27,28] teachers’ knowledge of diabetes is insufficient, this study aims to present a validated questionnaire for gathering information on teachers’ levels of knowledge of this disease.

Appropriate control of this condition can prevent the occurrence of complications related to elevated blood glucose. In particular, aspects such as the frequent monitoring of blood glucose that must be performed daily by the child, the administration of insulin either through multiple injections or with an infusion pump, the monitoring of physical activity and nutrition [29,30], and most importantly, hypoglycemia, which demands immediate care [31]. Such control should be addressed in the school environment, as children spend much of their day in school and the likelihood of experiencing complications from this disease is high [32].

It is essential that school staff is appropriately trained to provide safe and effective care to students with diabetes [33]. This is why new educational strategies must be developed to improve teachers’ knowledge of this disease, which is so widespread among children and adolescents.

The objective of this study was to present the content validation procedure of an instrument for determining the degree of knowledge of DM1 in school teachers through expert judgment, which will make it possible to design promotion, prevention, and health education plans for school teachers that will undoubtedly have an impact on the quality of life of schoolchildren with diabetes.

## 2. Materials and Methods

This study, which was conducted at the Melilla Campus of the University of Granada, Spain, used a descriptive, cross-sectional design to present the content validation procedure for a questionnaire to determine levels of knowledge of DM1 among school teachers using expert judgment and a pre-test.

### 2.1. Participants

The sample was selected using convenience (intentional) sampling, and consisted of 15 professionals from different areas of knowledge, with professional experience (in both teaching and conducting research) of between 5 and 30 years (SD: 19.4 years). They all voluntarily agreed to participate in the study by signing an informed consent form. Table 1 shows the areas of knowledge as well as the years of experience for each of the experts.

### 2.2. Instrument

The validation process was applied to a 33-item questionnaire built on the basis of the 24 items that make up the diabetes knowledge questionnaire (DKQ-24) [34], an instrument that was designed to assess general knowledge of diabetes in individuals in accordance with the content recommendations of the U.S. National Standards for Diabetes Patient Education Programs [35], plus 9 newly created items to complement the different aspects under study, such as knowledge of complications and knowledge of diabetic patient care (Appendix A). These 9 items were prepared based on the literature review conducted using the Web of Science database taking into account the classical test theory [36].

The questionnaire, “Grado de conocimientos sobre diabetes en docentes del ámbito escolar” (Spanish for “Level of knowledge of diabetes in school teachers”), henceforth GCDDaE, consists of 4 dimensions: general knowledge of the disease (14 items), knowledge of symptoms (5 items), knowledge of complications (5 items), and knowledge of diabetic patient care (9 items). The questionnaire was evaluated by assessing each item using the “expert judgment” template developed by Escobar-Pérez and Cuervo-Martínez [17]. These authors established four categories to be assessed (sufficiency, clarity, coherence, and relevance) using a scale with four options: does not meet the criterion, low level, moderate level, and high level (Table 2). In addition, also included was the possibility for experts to make qualitative observations on the accuracy of the language and content of each of the 33 items that made up the original instrument.

### 2.3. Procedure

After contacting the experts by telephone, the questionnaire to be validated was sent to them by e-mail together with the information necessary to make their evaluation as well as the informed consent form and our assurance that their data would remain confidential. The experts were given one month to assess and rate all the items. After this time, they returned their assessments and their signed informed consent form by e-mail. No reminders had to be sent to them.

Once the questionnaire had been evaluated by the experts, and taking into account the qualitative observations made, two items (belonging to the dimension “knowledge of diabetic patient care”) were removed from the original questionnaire because they failed to meet all the established requirements, resulting in a final instrument consisting of 31 items distributed in the same 4 aforementioned dimensions: general knowledge of the disease (14 items), knowledge of diabetic patient care (7 items), knowledge of symptoms (5 items), and knowledge of complications (5 items). 

Subsequently, the instrument was pre-tested using dichotomous yes/no responses in order to measure its degree of real-life feasibility and/or applicability and to assess the level of understandability of each item [37]. The pre-test was conducted with a random sample of 114 education students from the Faculty of Education and Sport Sciences at the Melilla Campus of the University of Granada, Spain, who had previously submitted their signed informed consent form. The percentages and levels of comprehensibility obtained with the data collected were classified into the following categories: high (equal to or greater than 85%), medium (from 80 to 85%), and low (less than 80%).

### 2.4. Ethical Considerations

All research participants were duly informed and voluntarily accepted to take part in the study by signing the corresponding informed consent, referring to the ethical principles of the Helsinki Declaration. The approval of the Ministry of Education and Vocational Training was obtained with reference: EA0022075s18N0002381 on 11 December 2018.

### 2.5. Statistical Analysis

Data were processed using the statistical package SPSS, version 24.0, for Windows. As the instrument contained a number of categories of an ordinal nature, Fleiss’ *κ* coefficient was used to assess the degree of agreement between the judges [18,38,39].

Fleiss’ *κ* values, which can range from 0 to 1, were interpreted using the classification created by Landis & Koch [40], as shown in Table 3, which expresses the strength of agreement between the judges.

Basic statistics (frequencies and percentages) were used to analyze the data collected in the pre-test.

## 3. Results

### 3.1. Content Validation through Expert Judgment

Table 4 shows the Fleiss’ *κ* values for the proportion of possible agreements occurring in each dimension, as well as the strength of agreement estimated using the classification created by Landis and Koch [40]. In this case, the agreement is considered to be almost perfect for three dimensions (“general knowledge of the disease”, “knowledge of symptoms”, and “knowledge of complications”) and substantial for the remaining dimension (“knowledge of diabetic patient care”).

Fleiss’ *κ* values by pairs of experts were also determined, which yielded a greater variability, between moderate and almost perfect, revealing some discordance, especially in the dimension of “knowledge of diabetic patient care”, as shown in Table 5.

In order to assess the characteristics of the instrument as a whole, the categorical indicators of sufficiency, clarity, coherence, and relevance were rated using an ordinal scale. The results revealed a strength of agreement between substantial and almost perfect, with “sufficiency” and “relevance” having the highest Fleiss’ *κ* values (0.982 and 0.903, respectively). Statistical significance was below 0.05 in all cases, with a 95% confidence. As shown in Table 6, the characteristics “sufficiency” (*p* < 0.001) and “relevance” (*p* = 0.001) were especially relevant.

This statistical procedure and its results, together with the qualitative observations and recommendations made by the judges for each item, produced the final validated instrument (Appendix A).

### 3.2. Measurement of Applicability: Pre-Test

The final validated instrument (Appendix A) was completed by a total of 114 education students, and therefore future educators, to determine the degree of comprehensibility of the dimensions and their corresponding items. All of them were valid. The analysis of the data (percentages of yes/no responses given by the participants) allowed us to establish the degree of understandability of the instrument in the highest range, with percentages of understanding equal to or higher than 85% for all items, as shown in Table 7. The overall understandability of the final validated questionnaire was 91.54%.

## 4. Discussion

This study presents the content validation through expert judgment of an instrument for determining the degree of knowledge of diabetes in school teachers. Several studies highlight that these professionals’ knowledge of different aspects regarding emergencies in individuals with diabetes, such as hypoglycemic and hyperglycemic events, is quite limited, making it difficult to safely and fully integrate children with diabetes into schools [22,27,28,41,42].

The design and construction of a measurement instrument is a complex process in which the aim is to define the objective, the references, the construct, and the dimensions in which the items are proposed [43]. This is not a disadvantage when it comes to obtaining an easily and quickly administered instrument with acceptable psychometric results both at a global level and at the level of each dimension and, of course, an instrument that is welcomed by professionals [44].

This study is based mainly on the process of transcription of the instrument, its validation, and the analysis of its psychometric properties. This process was structured following methodological designs that indicate in a stepwise manner how to explore the different psychometric aspects of the instrument, which provide statistical consistency and support that should be taken into account. More psychometric properties being determined across different contexts and subjects will result in a more consistent instrument. Content validation by expert judgment is the key part of the present study [7].

The content validity of a questionnaire is the extent to which its measurement represents the greatest number of dimensions of the concept to be studied. The content of an instrument is thus considered to be valid if it represents all the aspects related to the concept under study [45]. To avoid creating an overly broad, wide-ranging instrument and facilitate completion, a questionnaire should only include the most relevant, informative range of questions regarding the topic it is intended to address [43]. The questionnaire of our study captures, in 31 items, the main aspects needed to determine the level of knowledge of diabetes in school teachers.

This study used the expert judgment technique because this technique is reliable and relatively simple, and because it is frequently used in the content validation of instruments before administering them to the population [46]. However, difficulties are inevitable, such as the number of experts required to participate. In fact, in the literature consulted on this subject, the number of experts required differs considerably, and no consensus has been reached [37]. Delgado-Rico [47] recommend consulting at least three experts; a study by [10] involved eight experts, whereas other authors point out that the number of experts will depend on the objectives of the study, with a range of between seven and thirty judges [48]. In the present study, 15 experts were selected, which is approximately in line with the findings of the different authors consulted. With regard to experience, Escobar-Pérez and Cuervo-Martínez [17] recommend that the expert group should include, among others, measurement and assessment professionals. As a result, in this study, two judges from the field of research and diagnostic methods in education were included, as reported in a study by Fernández-Gómez et al. [18]. 

In addition, since the aim of this study was to validate the items of a questionnaire on diabetes (the GCDDaE questionnaire), six of the experts were nurses, who were selected for their connection with diabetes education. The judges’ wide variety of areas of knowledge turned out to be very positive, as it brought together different forms of expertise on the subject matter [49]. The quality and diversity of professionals may be more meaningful than their number [50]. 

The selection of the experts is another important consideration. Cabero and Llorente [37] list a number of selection procedures, including structured selection procedures, such as the proximity or affinity of the researchers to the judges, and unstructured selection procedures, such as graphical biographies and the expert competence coefficient. An unstructured selection procedure has been used in this study.

This type of validation, however, may involve significant errors. If the judges know what is intended to be measured and these constructs are defined by the researchers themselves, there is a risk of directing the experts’ assessments [51].

Nevertheless, if we wish to obtain advantageous results to achieve our main objective, we must pay particular attention to other characteristics in the selection of the judges [37]. According to [17], these other characteristics in the selection of the judges are their experience, their academic and scientific reputation, their availability, and their motivation to participate, but most importantly, their honesty and commitment regarding these characteristics. Judges with the aforementioned criteria participated in this study in order to avoid introducing content bias in the data analysis [36].

In addition, to ensure validity, in addition to the set of experts, it is very useful to include non-specialists, mainly individuals from the reference population [50]. In the present study, 114 university students from the Faculty of Education and Sport Sciences at the Melilla Campus of the University of Granada, Spain, all of them future school teachers, voluntarily participated in the pre-test, in a similar way to what [36] described in their study. As in the case of their study, the degree of comprehensibility of the GCDDaE questionnaire was found to be high (91.54%).

Fleiss’ *κ* was suitable for identifying and assessing the magnitude of the strength of agreement, which was almost perfect for each dimension and between moderate and almost perfect for pairs of experts, in line with the results obtained by Bernal-García et al. [36]. This coefficient is based on the statistic known as Cohen’s coefficient (*κ*), which calculates the probability of agreement as long as it is between two raters or observers [52]. However, to calculate the degree of agreement between three or more raters, Fleiss’ *κ* is used [53], which is the aim of the present study. This procedure offers the possibility of eliminating random chance agreement and provides quantifiable methods to assess observations on content [52]. Evaluating these properties is an essential criterion for determining the quality of their measurements [7].

Following the procedure used by [17], the analysis of each item was performed on the basis of four criteria: sufficiency, clarity, coherence, and relevance, with Fleiss’ *κ* coefficient confirming the applicability of the test. In addition, the qualitative analysis of the items made by the experts regarding the transcription of the items and the coherence, accuracy, and comprehensiveness of the content allowed us to modify and eliminate items without compromising the validity of the instrument [36]. In the present study, seventeen items were modified and two items were removed after the qualitative analysis of the experts. With respect to the degree of comprehensibility of the dimensions and their items in the final version of the validated instrument, it should be noted that the dimensions that were given the highest scores were those relating to the general knowledge of the disease and its potential complications, as opposed to the dimensions that were given the lowest scores, those relating to the knowledge of symptoms and the knowledge of diabetic patient care.

With regard to the implications for health professionals, it is worth noting the unavoidable need to disseminate guidelines for the creation of questionnaires, their consolidation, and their adaptation using content validation through expert judgment. This could be serve to develop new instruments that contribute to the practice and research of safe and reliable methods that help to improve the quality of life and safety of schoolchildren, also serving as indicators to guide the implementation of strategies, programs, and interventions in relation to students with DM1.

In the process of validation through expert judgment, there are aspects that researchers cannot fully control. As a result, our study is not devoid of limitations. However, although the results of the tests conducted for the validation of this instrument were acceptable, it is advisable to review the content validity of the instrument periodically.

Possible new lines of research derived from this study could be aimed at developing health education training programs in schools and training guidelines on the management of childhood diabetes for the educational community.

## 5. Conclusions

The results obtained using the Fleiss’ *κ* coefficient increased the objectivity of the instrument by adding an interesting quantitative value for measuring the degree of agreement between experts and enabling the researchers to validate the questionnaire correctly. To conclude with validity, the pre-test procedure helps to determine the degree of understandability of the original instrument. It is important for both healthcare professionals and researchers to understand the procedure for conducting a study on the content validation of an instrument. We believe it is advisable to use the GCDDaE questionnaire, as it is feasible and quickly administered, it has acceptable psychometric characteristics and has been approved by the experts both at the level of each dimension and at a general level. The questionnaire has a considerable potential to elucidate the degree of knowledge that teachers have and contribute to their training by increasing their knowledge of diabetes and, as a result, increase the quality of life and safety of schoolchildren with DM1.

## Figures and Tables

**Table 1:** Judges, areas of knowledge, and work experience.

Judge	Field of Expertise/Academic Training	Years of Work Experience
1	Language and Literature Didactics	29
2	Language and Literature Didactics	35
3	Health Sciences	5
4	Health Sciences	33
5	Health Sciences	23
6	Health Sciences	21
7	Health Sciences	6
8	Health Sciences	7
9	Research and Diagnosis Methods in Education	21
10	Research and Diagnosis Methods in Education	18
11	Personality, Evaluation, and Psychological Treatment	34
12	Personality, Evaluation, and Psychological Treatment	30
13	Didactics and School Organization	14
14	Nutrition and Food Science	6
15	Biochemistry and Molecular Biology II and Immunology	10

**Table 2:** Categories and indicators used by the judges to validate the tool.

Categories	Indicators
**Sufficiency**—The items within the same dimension suffice to measure this dimension	The items are sufficient to measure the dimension
	The items measure some aspects of the dimension, but do not represent the full dimension
	A few items must be added in order to fully assess the dimension
	The items are insufficient
**Clarity**—The item can be understood easily, i.e., syntax and semantics are appropriate	The item is unclear
	The wording of the item requires several modifications or a very large modification in terms of meaning or word order
	Some of the terms in the item require very precise modifications
	The item is clear, with appropriate semantics and syntax
**Coherence**—The item is logically related to the dimension or indicator it is measuring	The item bears no logical relationship to the dimension
	The item has a tangential relationship to the dimension
	The item has a moderate relationship to the dimension it is measuring
	The item is completely related to the dimension it is measuring
**Relevance**—The item is essential or important, i.e., it must be included	The removal of the item would not affect the measurement of the dimension
	The item is somewhat relevant, but another item may be covering what this item is measuring
	The item is rather important
	The item is very relevant and should be included

Source: adapted from Escobar-Pérez and Cuervo-Martínez (2008, p. 37).

**Table 3:** Fleiss’ *κ* values and strength of agreement (Landis and Koch, 1977).

Fleiss’ *κ*	Strength of Agreement
0	Poor
0.1–0.20	Slight
0.21–0.40	Fair
0.41–0.60	Moderate
0.61–0.80	Substantial
0.81–1.00	Almost perfect

**Table 4:** Strength of agreement among judges for the dimensions of the original instrument.

Dimensions	Fleiss’ *κ*	Strength of Agreement (Landis & Koch, 1977)
General knowledge of the disease (D1)	0.902	Almost perfect
Knowledge of symptoms (D2)	0.898	Almost perfect
Knowledge of complications (D3)	0.873	Almost perfect
Knowledge of diabetic patient care (D4)	0.791	Substantial

**Table 5:** Agreement by pairs of experts.

Dimensions	Fleiss’ *κ*—Agreement by Pairs of Experts														
	1–15	2–14	3–13	4–12	5–11	6–10	7–9	8–7	9–6	10–5	11–4	12–3	13–2	14–1	15–8
D1	0.929	0.868	0.763	0.885	0.717	0.920	1	0.830	0.811	0.785	0.900	0.735	0.889	1	1
D2	1	0.984	0.711	0.700	0.732	0.833	0.931	0.846	0.744	0.714	0.706	0.708	0.949	1	1
D3	1	0.844	0.811	0.790	0.832	0.893	0.931	0.746	0.944	0.814	0.906	0.850	0.849	1	1
D4	0.812	0.784	0.811	0.732	0.712	0.633	0.753	0.646	0.674	0.814	0.656	0.718	0.749	1	0.892

**Table 6:** Fleiss’ *κ* coefficient and statistical significance of the characteristics of the original instrument.

Characteristics	Fleiss’ *κ*	*p*
Sufficiency	0.982	0
Clarity	0.792	0.024
Coherence	0.812	0.016
Relevance	0.903	0.001

**Table 7:** Percentages of comprehensibility of the dimensions and their items in the final version of the validated instrument.

Dimension	Item	Degree of Comprehensibility (%)	
		Yes	No
General knowledge of the disease (D1)	Insulin	95.5	3.5
	Types of diabetes	97.3	2.7
	Relationship between food intake and insulin	93.8	6.2
	Medication versus diet	92.9	7.1
	Insulin production	91.2	8.8
	Heredity and diabetes	96.5	3.5
	Glycemia and treatment	94.7	5.3
	Cure for diabetes	93.8	5.2
	Blood sugar levels	86.7	13.3
	Diabetes control	96.5	3.5
	Physical activity and diabetes	88.5	10.5
	Cause of diabetes: kidney filtering failure	95.6	4.4
	Cause of diabetes: insulin	96.5	3.5
	Cause of diabetes: intake of sugar and sweets	94.7	5.3
Knowledge of symptoms (D2)	Frequent urination and thirst	93.8	6.2
	Trembling and sweating	93.8	6.2
	Hypoglycemia	87.6	12.4
	Hyperglycemia	85.0	15.0
	Diabetes in children	85.0	15.0
Knowledge of complications (D3)	Kidney damage	96.5	3.5
	Blood circulation	97.3	2.7
	Loss of sensation in extremities	94.7	5.3
	Wound healing	93.8	6.2
	Cuts	89.4	10.6
Knowledge of diabetic patient care (D4)	Food preparation	85.0	15.0
	Special foods	90.3	9.7
	Cleaning of wounds	92.9	7.1
	Capillary blood glucose monitoring	87.6	12.4
	Insulin injection site	91.2	8.8
	Insulin temperature	88.5	11.5
	Inclination of the needle	87.6	12.4

## Appendix A

**Table A1:** Level of Knowledge of Diabetes.

		True	False	I Don’t Know
1	Diabetes is caused by having a sufficient amount of a hormone called insulin.			
2	There are two main types of diabetes: type 1 (insulin-dependent) and type 2 (non-insulin-dependent).			
3	Increases in blood insulin are caused by the intake of large amounts of food.			
4	Pharmacological treatment is more important in controlling diabetes than diet and exercise.			
5	Insulin is produced in the kidneys.			
6	A person with diabetes is more likely to have children with diabetes.			
7	In untreated diabetes, the level of blood glucose usually increases.			
8	Diabetes can be cured.			
9	A fasting blood glucose level of 210 (mg/dL) is very high.			
10	The best way to control diabetes is through urine tests.			
11	Regular exercise will increase the need for insulin or other diabetes medication.			
12	Diabetes often causes poor blood circulation.			
13	How food is prepared (fried, roasted, etc.) is as important as the food that is consumed.			
14	Diabetes can cause damage to the kidneys.			
15	A diet for people with diabetes consists mainly of special foods.			
16	Eating too much sugar and other sweet foods is one of the causes of diabetes.			
17	The common cause of diabetes is a lack of effective insulin in the body.			
18	Diabetes is caused because the kidneys cannot filter the sugar from the blood.			
19	Frequent urination and thirst are signs of low blood glucose.			
20	Trembling and sweating are signs of low blood glucose.			
21	Wounds and scratches heal more slowly in people with diabetes.			
22	People with diabetes have to be extra careful when clipping their toenails.			
23	An individual with diabetes should clean the wound first with povidone-iodine and then with alcohol.			
24	Diabetes can cause a loss of sensation in the hands, fingers, and feet.			
25	The main symptoms that children with diabetes may have are polydipsia, polyphagia, polyuria, and anorexia, among others.			
26	In the case of hypoglycemia with loss of consciousness, juice, sweets, sugar, etc. should be administered.			
27	Capillary blood glucose monitoring is done with a finger prick, on the fingertip if possible, which is the least pain-sensitive area of the finger.			
28	The area and the injection site of the insulin must be changed weekly.			
29	Cold insulin hurts less and is better absorbed.			
30	The inclination of the needle on the skin when punctured should always be 90°.			
31	Odd behavior and slurred speech are symptoms of hyperglycemia.			
